# Cardiac management during adjuvant trastuzumab therapy: recommendations of the Canadian Trastuzumab Working Group

**DOI:** 10.3747/co.2008.199

**Published:** 2008-01

**Authors:** J.R. Mackey, M. Clemons, M.A. Côté, D. Delgado, S. Dent, A. Paterson, L. Provencher, M.B. Sawyer, S. Verma

**Keywords:** Early-stage breast cancer, trastuzumab, cardiotoxicity, anthracycline, adjuvant chemotherapy

## Abstract

Trastuzumab has been shown to be an effective therapy for women with breast cancer that overexpresses the human epidermal growth factor receptor 2 (her2) protein. In the pivotal metastatic breast cancer trials, cardiac dysfunction was observed in women treated with trastuzumab and chemotherapy. The incidence and severity of cardiac dysfunction was greatest among patients who received trastuzumab in combination with anthracycline-based therapy. Those findings influenced the design of subsequent trastuzumab trials to include prospective evaluations of cardiac effects and protocols for cardiac monitoring and management. The risk of cardiotoxicity has also driven efforts to develop non-anthracycline-based regimens for women with her2-positive breast cancers.

With the increasing use of trastuzumab, particularly in the curative adjuvant setting, the need for a rational approach to the treatment and cardiac management of the relevant patient population is clear. The mandate of the Canadian Trastuzumab Working Group was to formulate recommendations, based on available data, for the assessment and management of cardiac complications during adjuvant trastuzumab therapy. The panel formulated recommendations in four areas:

- Risk factors for cardiotoxicity
- Effects of various regimens
- Monitoring
- Management

The recommendations published here are expected to evolve as more data become available and experience with trastuzumab in the adjuvant setting grows.

## 1. INTRODUCTION

Breast cancer is the most common female malignancy in the world 1. Globally, it accounts for 7% of all cancer- related deaths and 22% of all new cancer diagnoses in women. In Canada, breast cancer is similarly the most common cancer in women, with more than 22,000 new diagnoses every year. Breast cancer is responsible for the deaths of more than 5000 Canadian women annually, more than any other malignancy except lung cancer 2.

Approximately 20%–25% of breast cancers overexpress or amplify human epidermal growth factor receptor 2 (her2) 3,4. This cell-surface protein, which is involved in normal cellular growth and differentiation, is a member of the her (ErbB) family of transmembrane receptor tyrosine kinases. Tumours that overexpress the her2 protein or that amplify the *HER2*/*neu* gene are associated with an aggressive disease course and a poor prognosis, with high risk of recurrence and metastasis 3–6.

Trastuzumab, a humanized monoclonal antibody, was developed to target her2 7. When used in combination with taxanes in the first-line treatment of metastatic breast cancer, trastuzumab improves survival and quality of life 8–10. Trastuzumab has also shown efficacy as monotherapy in metastatic breast cancer, both as first-line treatment and in patients whose tumours failed to respond to one or more chemotherapies 11–13.

Motivated by the considerable antitumour effect of trastuzumab in her2-positive metastatic breast cancer, four major international studies of trastuzumab in the adjuvant setting were initiated during 2000–2001. In Canada, early results of those trials led to the approval, in 2006, of trastuzumab for the adjuvant treatment of her2-positive breast cancer 14. Results of the trastuzumab adjuvant trials are summarized later in this paper.

During the pivotal metastatic breast cancer trials of trastuzumab, an unexpected toxicity—cardiac dysfunction—was observed in women treated with trastuzumab and chemotherapy 10. The incidence and severity of cardiac dysfunction was greatest among patients who received trastuzumab in combination with an anthracycline. Those findings influenced the design of subsequent trastuzumab trials to include prospective evaluations of cardiac effects and cardiac monitoring and management protocols. They also drove efforts to develop non-anthracycline treatment regimens for her2-positive breast cancers 15,16.

With the increasing use of trastuzumab, particularly in the curative adjuvant setting, the need for a rational approach to the treatment and cardiac management of the relevant patient population is clear. The mandate of the Canadian Trastuzumab Working Group was to formulate, based on available data, recommendations for cardiac management during adjuvant trastuzumab therapy. The recommendations published here are expected to evolve as more data become available and as experience with trastuzumab in the adjuvant setting grows.

## 2. DEVELOPMENT OF RECOMMENDATIONS

The Canadian Trastuzumab Working Group (seven oncologists and two cardiologists) met in Toronto for a full-day conference in March 2007. The panel reviewed results of the adjuvant trastuzumab trials, as well as the cardiac parameters used in those trials—including cardiac eligibility criteria, definitions of cardiac effects, monitoring protocols, and management strategies and results. Based on that information, recommendations were formulated for monitoring and management of cardiac side effects during adjuvant trastuzumab therapy. An outline—and then a draft manuscript of the recommendations —as reviewed by three panel members, and the full manuscript was reviewed by all panel members. Recent clinical trial results (released since March 2007) were incorporated into the present document. The recommendations discussed here will be updated as additional evidence becomes available.

Development of the recommendations and the present manuscript were funded by an unrestricted educational grant from Roche Canada. The authors are responsible for the recommendations and the content of the manuscript, with no restrictions set by the sponsor.

The recommendations are made in four sections:

- Risk factors for cardiotoxicity
- Effects of various regimens
- Monitoring
- Management

Each section begins with a bulleted list of key recommendations, followed by a discussion of the available evidence, of the panel’s rationale for the recommendations, and of any points on which consensus was lacking.

## 3. THE TRASTUZUMAB ADJUVANT TRIALS

Four major adjuvant trials—hera (the Herceptin Adjuvant trial), the National Surgical Adjuvant Breast and Bowel Project (nsabp) B-31 trial, the North Central Cancer Treatment Group (ncctg) N9831 trial, and the Breast Cancer International Research Group (bcirg) 006 trial—investigated various adjuvant approaches with trastuzumab. Between them, these trials enrolled more than 13,000 women with her2-positive early breast cancer 17 (Table I). Results indicated that adjuvant trastuzumab reduces the 3-year risk of recurrence by nearly half in such patients. The benefit was similar across the trials despite differences in patient populations, chemotherapy regimens, and sequencing of treatment 17. In addition, a significant survival benefit was seen across all trials (Table II).

Based on the data from these pivotal adjuvant trastuzumab trials demonstrating significant disease-free survival and overall survival benefits, trastuzumab was adopted as the standard of care in her2-positive early breast cancer.

Comparisons between studies of trastuzumab-related cardiotoxicity are difficult. The studies used different entry criteria for cardiac function and cardiovascular risk, different definitions of cardiac dysfunction, and different parameters to assess cardiac safety. Nonetheless, each of the major trials showed a clear signal for increased cardiotoxicity with adjuvant trastuzumab. In absolute terms, the increased risk for New York Heart Association (nyha) grades III and IV heart failure (see Appendix A) was 0.4%–3.3%; in relative terms, risk increased by a factor of 5–10. The rate of asymptomatic decline by more than 10% in left ventricular ejection fraction (lvef) ranged from a high of 18% in bcirg 006 to a low of 3% in hera (Tables III and IV)^18^ ,^20^,^24^–^27^.

It is also clear that, although symptomatic heart failure may respond to heart failure medications 15, the drop in lvef in trastuzumab-treated patients does not necessarily fully recover to baseline. Although it is reassuring that, in these trials, no deaths from heart failure have occurred in trastuzumab-treated patients to date, the long-term consequences of the identified decreases in lvef, both symptomatic and asymptomatic, are as yet largely unknown. The syndrome of trastuzumab-associated cardiotoxicity exhibits clinical features that differ from those of classic anthracycline- associated cardiotoxicity (see Appendix B).

### 3.1 The HERA Trial

The hera trial compared 1 or 2 years of trastuzumab with observation alone in patients with her2-positive breast cancer who had completed locoregional therapy and at least 4 cycles of neoadjuvant or adjuvant chemotherapy 32. Treatment with trastuzumab for 1 year following adjuvant chemotherapy was associated with a significant overall survival benefit after a median follow-up of 2 years. The unadjusted hazard ratio for risk of death with trastuzumab as compared with risk of death with observation alone was 0.66 [95% confidence interval (ci): 0.47 to 0.91; *p* = 0.0115] 18.

The incidence of cardiac endpoints was higher in the trastuzumab group than in the observation group: severe heart failure (nyha grades iii and iv) was 0.60% as compared with 0.00%; symptomatic heart failure (including nyha grade ii heart failure) was 2.15% as compared with 0.12%; and confirmed significant decline in lvef was 3.04% as compared with 0.53% 24. No evidence of cumulative cardiotoxicity beyond 1 year was observed 18 ,32 . Most patients with cardiac dysfunction experienced symptomatic improvement and at least partial recovery of lvef less than 6 months after withdrawal of trastuzumab and initiation of medical treatment with angiotensin converting-enzyme (ace) inhibitors and beta-blockers 24.

The authors of a recent detailed analysis of cardiac endpoints in the hera trial suggested that the benefit of trastuzumab continues to increase into the 2nd year of follow-up, while the cumulative incidence of any type of cardiac endpoint appears stable after completion of trastuzumab at 12 months 24. However, they also acknowledge that the median follow-up in their report is only 12 months. Longer-term data will be needed to confirm their findings.

In terms of risk factors for trastuzumab-associated cardiotoxicity in the hera trial, patients who developed heart failure were treated with higher cumulative doses of doxorubicin (287 mg/m^2^ vs. 257 mg/m^2^) or epirubicin (480 mg/m^2^ vs. 422 mg/m^2^), and had a lower lvef (55%–60% vs. ≥60%, and 60%– 65% vs. ≥65%) and a higher body mass index (> 25 vs. 20–25) at screening. No associations were found between cardiac endpoints and older age, previous cardiac disease, hyperlipidemia, or hypertension. The investigators caution that analyses of potential risk factors are exploratory and based on a small number of cardiac events 24.

### 3.2 Joint Analysis: nsabp B-31 and ncctg 9831

The nsabp B-31 trial compared doxorubicin and cyclophosphamide followed by paclitaxel every 3 weeks (ac→p, group 1) with the same regimen plus 52 weeks of trastuzumab beginning with the first dose of paclitaxel (ac→ph, group 2). The ncctg N9831 trial compared three regimens: doxorubicin and cyclophosphamide followed by weekly paclitaxel (ac→p, group a), the same regimen followed by 52 weeks of trastuzumab after the paclitaxel (ac→p→h, group b), and the same regimen plus 52 weeks of trastuzumab initiated concomitantly with the paclitaxel (ac→ph, group c). The studies were amended to include a joint analysis comparing group 1 plus group a (the control group) with group 2 plus group c (the trastuzumab group). Group b was excluded from the joint analysis because trastuzumab was not given concurrently with paclitaxel 25.

An interim joint efficacy analysis of these trials showed that the absolute difference in disease-free survival between the trastuzumab group and the control group was 12% at 3 years. Trastuzumab therapy was associated with a 33% reduction in the risk of death (*p* = 0.015) 25. Following publication of the report, patients who had been randomized to ac→p and who were less than 6 months from completion of their chemotherapy were made eligible to receive adjuvant trastuzumab 19.

A recent efficacy update showed 4-year disease-free survival rates of 85.9% in the trastuzumab group and 73.1% in the control group (*p* < 0.00001) 19. Overall survival rates at 4 years were 92.6% and 89.4% respectively (*p* = 0.0007). The investigators concluded that the benefit of ac→ph is maintained with longer follow-up. The hazard of disease recurrence is reduced by 52% (*p* < 0.00001) and the hazard of death is reduced by 35% (*p* = 0.0007), despite the fact that 21% of patients randomized to the control group received trastuzumab.

An initial assessment of cardiac dysfunction in these trials showed that the 3-year cumulative incidence of cardiac events (grades iii and iv heart failure or cardiac death) was 4.1% in the trastuzumab group and 0.8% in the control group in nasbp B-31 and 2.9% with trastuzumab and 0% in the control group in ncctg N9831 25,33. With 2 years of additional follow-up, the cumulative incidence of cardiac events in the trastuzumab group in nsabp B-31 remained essentially unchanged at 3.8% (Table V) 26,34. However, 14.2% of patients had to stop trastuzumab because of an asymptomatic decline in lvef, and a total of 18.9% discontinued trastuzumab because of cardiac problems (Table III). These analyses also excluded patients who had already developed cardiotoxicity from anthracyclines at the completion of their 4 cycles of ac chemotherapy (6.7% of the initial group) and who were therefore ineligible to receive adjuvant trastuzumab. The absolute rate of cardiotoxicity in an intent-to-treat analysis would be somewhat higher.

### 3.3 bcirg 006

The bcirg 006 trial compared adjuvant doxorubicin and cyclophosphamide followed by docetaxel (ac→t) to the same regimen with trastuzumab added concurrently with the docetaxel (ac→th) and to docetaxel, carboplatin, and trastuzumab (tcbh). (Trastuzumab was given for 1 year on both arms that used it.) Results of the second interim analysis showed that 3-year disease-free survival rates were 77% for ac→t, 83% for ac→th, and 82% for tcbh (ac→th vs. ac→t, *p* < 0.0001; tcbh vs. ac→t, *p* = 0.0003). Overall survival rates were 86% for ac→t, 92% for ac→th, and 91% for tcbh (ac→th vs. ac→t, *p* = 0.004; tcbh vs. ac→t, *p* = 0.017) 20. This was the third trial to report significant disease-free survival and overall survival benefits.

No cardiac deaths were observed after 3 years of follow-up in this trial. Patients with grades iii and iv heart failure numbered 4 on the ac→t arm (of 1050), 20 on the ac→th arm (of 1068), and 4 on the tcbh arm (of 1056), with a *p* value of 0.0015 for ac→th vs. tcbh. The percentage of patients with a greater-than- 10% relative decline in lvef was 10% on the ac→t arm, 18% on the ac→th arm, and 8.6% on the tcbh arm (all differences statistically significant) 20.

### 3.4 The Finland Herceptin Trial

A Finnish trial, Finher, much smaller in scope than the four international adjuvant trials, treated women with early breast cancer with 3 cycles of docetaxel or vinorelbine, followed by 3 cycles of 5-fluorouracil, epirubicin, and cyclophosphamide. A subgroup of 232 patients with her2-positive breast cancer were further randomized to 9 weekly trastuzumab infusions 35.

Within the subgroup of her2-positive patients, women who received trastuzumab had a better rate of 3-year recurrence-free survival than women who did not receive trastuzumab (89% vs. 78%). No difference in overall survival was observed. Trastuzumab was not associated with decreased lvef or cardiac failure 35.

## 4. RECOMMENDATIONS FOR ASSESSMENT AND MANAGEMENT OF CARDIAC COMPLICATIONS DURING ADJUVANT TRASTUZUMAB THERAPY

Each subsection begins with a list of key recommendations. A discussion of the available evidence and the panel’s rationale for the recommendations then follows.

### 4.1 Risk Factors for Cardiotoxicity

- Risk factors that exclude patients from treatment with trastuzumab are existing heart failure or lvef < 50% [or below the facility’s lower limit of normal (lln)], or both.
  - Trastuzumab may be considered in patients with a lvef < 50% if their risk of disease recurrence is very high.
- Risk factors that require special consideration include
  - ischemic heart disease or significant valvulopathy,
  - a baseline lvef 50%–55% before trastuzumab therapy,
  - a decrease in lvef of more than 15% while on trastuzumab therapy, even if lvef remains above the lln 27.

#### Discussion

Cardiac eligibility criteria were similar, but not identical, across the four major adjuvant trials. The nsabp B-31, ncctg 9831, and bcirg 006 trials all required patients to have a lvef ≥ lln (50%) and no past or active cardiac disease (including myocardial infarction, heart failure, cardiomyopathy, angina pectoris or arrhythmia requiring medication, severe conduction abnormality, clinically significant valvular disease, uncontrolled hypertension, ventricular hypertrophy, or cardiomegaly on chest radiograph) 20,25.

Similarly, hera excluded patients with a history of cardiac disease 24. However, the acceptable lower limit for lvef was higher in hera, at ≥55%. Patients enrolled in hera also received trastuzumab *after* completing chemotherapy; patients reported in the other three trials had been enrolled with normal cardiac function (lvef ≥ 50%) *before* receiving any systemic therapy for breast cancer. The cardiac-specific report on hera suggests that these factors may have contributed to the lower incidence of cardiac dysfunction in the hera trial. That is, patients who experienced a drop in lvef following anthracycline-based chemotherapy would not have been eligible for the hera trial 24.

The nsabp B-31 5-year update identified four risk factors for heart failure in trastuzumab-treated patients 26:

- Age (50–59 years, 5.1%; ≥60 years, 5.4%)
- Use of hypertensive medications (6.8%)
- Baseline lvef values of 50%–54% (12.9%)
- Post-anthracycline chemotherapy lvef values of 50%–54% (12.6%)

The number of patients over 60 years of age in that trial was 148; the number on hypertensive medications was 192; the number that had a baseline lvef < 54% was 70; and the number that had a post-anthracycline lvef < 54% was 111.

The hera trial results also showed that a lower baseline lvef value and a high body mass index are risk factors for heart failure in trastuzumab-treated patients. However, in contrast to nsabp B-31, hera observed no association between cardiac endpoints and increased age, previous cardiac disease, hyperlipidemia, or hypertension 24.

Both hera and ncctg N9831 investigated the use of radiotherapy in trastuzumab-treated patients. In the hera trial, previous radiotherapy did not increase the incidence of cardiac dysfunction 24. In ncctg N9831, concurrent radiotherapy and trastuzumab did not increase the incidence of cardiac events or radiotherapy-associated adverse events, with the exception of leucopenia 36. The investigators cautioned that longer follow-up is required to determine whether any longterm cardiotoxicity emerges.

### 4.2 Effects of Various Regimens on Cardiotoxicity

- The data are insufficient to make any formal statements regarding the relative cardiotoxicity of concurrent as compared with sequential treatment regimens, or of anthracycline use of in patients with cardiac risk factors.
- On the basis of hera and of the ncctg N9831 comparison of group b (ac→t→h) and group c (ac→th), sequential anthracycline–taxane–trastuzumab regimens may *possibly* be less cardiotoxic than concurrent regimens that include anthracyclines—that is, anthracycline–taxane+ trastuzumab).
- Direct randomized evidence, as reported in the bcirg 006 trial, suggests that the anthracycline-free tcbh regimen has a rate of severe cardiotoxicity that is one fifth that of the anthracycline- containing ac→th regimen (incidence of grades iii and iv heart failure 0.4% vs. 1.9%) 20.
- Until such time as optimal duration of trastuzumab therapy has been established by ongoing adjuvant trials [phare (Protocol of Herceptin Adjuvant with Reduced Exposure), hera, and the Danish Finher replacement study], patients with early breast cancer should be treated with trastuzumab for 1 year (less only if disease recurs) 14.

#### Discussion

The hera trial reported a low incidence (0.6%) of severe heart failure despite the use of anthracycline-based chemotherapy in most patients. That finding may be the result of contributions from two principle mechanisms:

- Selection of patients with a lvef ≥ 55% after adjuvant chemotherapy (the incidence of heart failure in the hera trial patients was reduced relative to other trials that used a pre-chemotherapy lvef cut-off of 50%)
- Timing of trastuzumab administration (trastuzumab was typically administered some months after completion of anthracycline chemotherapy) 18,24

However, differences between the trial designs make this comparison informal at best.

The only adjuvant trial that directly compared concurrent with sequential trastuzumab treatment is the ncctg N9831 trial. Two preliminary reports on the efficacy and cardiotoxicity of concurrent versus sequential therapy have been published. One report compared group b (ac→t→h) with group c (ac→th) and suggested that sequential treatment may be less effective than concurrent treatment (Perez PA, Suman VJ, Davidson N, Martino S, Kaufman P. Advances in monoclonal antibody therapy for breast cancer: further analysis of NCCTG-N9831. Presented at the 41st Annual Meeting of the American Society of Clinical Oncology; May 13–17, 2005; Orlando, Florida). However, that interim analysis (requested by the data monitoring committee) had low statistical power; longer follow-up is needed to definitively address the question. The planned cardiac safety analysis of ncctg N9831 showed that the incidence of grades iii and iv heart failure was 3.3% with concurrent treatment and 2.2% with sequential treatment 37.

Results from the bcirg 006 trial suggest that the tcbh regimen is less cardiotoxic, both acutely and with 3-year follow-up, than the ac→th) combination 20. These data are the only available randomized comparison of an anthracycline-based with a non-anthracycline-based trastuzumab regimen, and they demonstrate a rate of grades iii and iv heart failure (20 patients, 1.9%) that is higher by a factor of 5 with ac→th than with tcbh (4 patients, 0.4%). Panel members favoured the non-anthracycline tcbh regimen for patients with pre-existing risk factors for cardiotoxicity.

The Finher trial treated 232 patients with trastuzumab for only 9 weeks. Analysis showed a relapse-free survival benefit and no clinical cardiotoxicity, but no significant difference in overall survival at 3 years 35. The hera trial includes a third arm in which patients are receiving 2 years of trastuzumab 32. The first results are expected later in 2008. The panel agreed that until such time as data become available regarding either shorter or longer durations of therapy, early breast cancer patients should be treated with trastuzumab for 1 year (less only if disease recurs). That decision accords with the product monograph 14, the major adjuvant clinical trials, and the major clinical guidelines (Cancer Care Ontario 38, British Columbia Cancer Agency 39, National Comprehensive Cancer Network 40, Comité de l’évolution des pratiques en oncologie 41, and St. Gallen’s 42). The guidelines also all indicate that trastuzumab is the standard of care for patients with node-positive disease or node-negative disease with a tumour size larger than 1 cm, regardless of hormone receptor status.

### 4.3 Monitoring

Given the relatively short follow-up times of the adjuvant trastuzumab trials and the incomplete recovery of cardiac function seen in those trials, even when heart failure medications are used, the panel emphasized the need for careful selection of patients, and consistent cardiac monitoring at 3-month intervals during the 1-year period of trastuzumab therapy.

- Assessment of cardiac function per established protocols is critical and must be endorsed for all patients.
- Either echocardiography or multiple gated acquisition scan should be used to establish baseline lvef. The same imaging modality should be used at follow-up.
  - Multiple gated acquisition scanning is generally more widely available in Canada and may be subject to less variability.
  - If echocardiography is used, the same technique must be used for each assessment. The preferred technique is the Simpson method.
- The lvef should be assessed before trastuzumab treatment is started (and after chemotherapy, for sequential regimens) and should be repeated every 3 months until completion of trastuzumab therapy. Each patient will therefore undergo a minimum of 5 lvef assessments: immediately before trastuzumab is initiated and at 3, 6, 9, and 12 months in the course of therapy.
  - Patients who experience cardiac symptoms or a greater than 10% absolute asymptomatic decline in lvef while receiving trastuzumab may continue to undergo annual cardiac assessments following completion of trastuzumab treatment.
  - At this time, no evidence exists to support further cardiac monitoring of patients who have completed chemotherapy and trastuzumab treatment with no cardiac symptoms and no signs of substantial (greater than 10% absolute decrease), but asymptomatic, lvef decline.
- The cardiac monitoring requirements outlined in the present article should be understood to represent the *minimum* monitoring requirements. Patients with cardiotoxicity or other risk factors may require more frequent and more stringent monitoring.

#### Discussion

As new therapies move from phase iii clinical trials to the clinic, patient selection criteria and monitoring generally become more flexible. Patients who would not have been eligible for clinical trials may be offered treatment, whether in hope of a cure or because of a lack of other options. Monitoring often becomes more infrequent and irregular without the standardized requirements of trial-mandated protocols. Panel members reported anecdotally that, in their tertiary care centres, cardiac monitoring rates of trastuzumab patients not enrolled in trials have not always been optimal.

To ensure patient safety and optimal treatment, rigorous monitoring of trastuzumab patients is of paramount importance. The adjuvant trials have shown that patients can experience a significant decline in lvef without experiencing symptoms. The lvef assessment schedule recommended here is based on schedules used in the adjuvant trials. The trial schedules varied somewhat with the regimens, but the basic approach was to assess cardiac function every 3 months during therapy.

The panel was divided on the need for continued monitoring of asymptomatic patients with a normal lvef after the completion of trastuzumab treatment. Some panel members felt that annual assessments of these patients would be advisable until more is known about the long-term cardiac effects of trastuzumab. Others felt that such monitoring could place an undue burden on patients and the health care system alike, and also perhaps expose patients to unnecessary doses of radiation. Further long-term data are needed to clarify this issue.

### 4.4 Management of Cardiotoxicity

- Management of trastuzumab-related cardiotoxicity has two distinct aspects: withdrawal of trastuzumab therapy and treatment of cardiac dysfunction.
- The “stopping/restarting” rules used in the adjuvant trials were effective and are recommended (Table VI), with some modifications regarding recommendations for a cardiology consult or treatment of cardiac dysfunction (or both) when appropriate.
- Symptomatic left ventricular (lv) dysfunction must be treated per Canadian Cardiovascular Society recommendations for heart failure treatment 21:
  - All patients with heart failure and a lvef below 40% should be treated with an ace inhibitor in combination with a beta-blocker unless a specific contraindication exists (class i, level a evidence).
  - Some members of the panel also felt that, to prevent further degradation of lvef or the development of clinical heart failure, an ace inhibitor should be considered if the patient’s lvef is between 40% and 50%.
- Asymptomatic lv dysfunction should be treated per the recommendations of the Canadian Cardiovascular Society 43:
  - ace inhibitors should be used in all asymptomatic patients with lv dysfunction and an ejection fraction below 40% (class i, level a evidence for ejection fraction below 35%; class i, level b for ejection fraction between 35% and 40%).
  - Some members of the panel also felt that an ace inhibitor should be considered if lvef is below 50%.
  - Beta-blockers should be considered in all patients with asymptomatic lv dysfunction and a lvef below 40% (if prior myocardial infarction, class i, level b evidence; if no myocardial infarction, class iia, level c).
- Initiation of pharmacotherapy for trastuzumab-related cardiotoxicity must be carried out on an accelerated schedule, because the normal titration schedules can take several months to reach the optimal therapeutic dosage (Table VII).
- The data are insufficient to make a definitive recommendation regarding duration of treatment for cardiac dysfunction in trastuzumab patients (see the Discussion). The duration of treatment with cardiac medication must be individualized.
- Following withdrawal of trastuzumab therapy because of cardiac dysfunction, trastuzumab may be re-initiated on the basis of the same lvef guidelines as the original initiation of therapy.

#### Discussion

Management of trastuzumab-related cardiotoxicity has two distinct aspects: withdrawal of trastuzumab therapy and treatment of cardiac dysfunction. The “stopping/restarting” rules used in the adjuvant trials were effective. The panel recommends the nsabp B-31 protocol (Table VII), with some modifications to include recommendations for a cardiology consult or treatment of cardiac dysfunction (or both) when appropriate.

Treatment of trastuzumab-related cardiotoxicity is a controversial subject. Patients who developed lv dysfunction in the adjuvant trastuzumab trials were not treated in a systematic manner. Trastuzumab-related cardiotoxicity does seem to be partially reversible when trastuzumab is withdrawn and medical therapy is initiated for symptomatic heart failure 15. The panel recommended withdrawal of trastuzumab and initiation of medical therapy for these reasons:

- Standard therapy for lv dysfunction and heart failure may hasten recovery after withdrawal of trastuzumab 44.
- Because of the longer life expectancy of patients with early-stage breast cancer, consideration of the potential long-term cardiotoxicity of trastuzumab is imperative. At this time, the longest reported adjuvant trastuzumab experience is only 4 years 20,26. The median reported follow-up in hera is 2 years, and in bcirg 006 and the joint analysis, it is 3 years.
- Beta-blockers and ace inhibitors have been shown to improve survival in heart failure arising from many causes; they are the cornerstone of heart failure therapy 21,43.

The question of duration of therapy for cardiotoxicity is also controversial and may not be resolved for some time. Cardiology guidelines support continued use of therapy after a diagnosis of lv dysfunction 21,43. However, oncologists may be reluctant to put relatively young and otherwise healthy women on lifelong therapy for what may be a short-term or self-limiting side effect of cancer therapy. More longterm data are needed to clarify this issue.

In the absence of long-term data on the natural history of trastuzumab-associated cardiotoxicity, the duration of therapy is left to the judgment of the treating clinician (oncologist, cardiologist, family practitioner) and may be determined by factors such as the degree of lv dysfunction, patient preference and symptoms, and degree of functional recovery.

## 5. CONCLUSIONS

The benefits of trastuzumab in her2-positive early breast cancer are well established. In early breast cancer, trastuzumab reduces the 3-year risk of recurrence by nearly half and the risk of death by one third. With the use of trastuzumab increasing, the need to optimize the approach to treatment and management of trastuzumab-related cardiotoxicity is clear. The mandate of the Canadian Trastuzumab Working Group was to formulate recommendations, based on available data, for cardiac management during adjuvant trastuzumab therapy. The recommendations set out here are expected to evolve as more data accrue and experience with trastuzumab in the adjuvant setting grows.

## 6. GUIDELINE DATE

This guideline was completed December 11, 2007.

## Figures and Tables

**TABLE I:** Trastuzumab (h) adjuvant trials: patient characteristics

Patient characteristics	hera^18^		Joint analysis^19^ (ncctg N9831, nsabp B-31)		bcirg 006^20^		
	Observation (%)	h (%)	Control (%)	h (%)	ac→t (%)	ac→th (%)	tcbh (%)
Age							
<50 years	52	52	51	51	52	52	54
≥50 years	48	48	49	49	48	48	46
er+ or pr+ (or both)	50	50	53	52	54	54	54
Nodal status							
Node-negative	33	32	13	11	29	29	29
1–3 Nodes positive	29	29	52	54	38	38	39
≥4 Nodes positive	28	28	41	41	33	33	33
Tumour size							
≤2 cm	40	40	38	41	38	40	
>2 cm	49	58	61	59	62	60	

hera = Herceptin Adjuvant Trial; ncctg = North Central Cancer Treatment Group; nsabp = National Surgical Adjuvant Breast and Bowel Project; bcirg = Breast Cancer International Research Group; ac = doxorubicin–cyclophosphamide; t = docetaxel; er+ = estrogen receptor– positive; pr+ = progesterone receptor–positive.

**TABLE II:** Trastuzumab (h) adjuvant trials: efficacy

Trial		Patients (n)	Disease-free survival (%)		Overall survival (%)	
			h arm	Non-h arm	h arm	Non-h arm
hera^18^(2-Year median follow-up)		5102	80.6	74.3	92.4	89.7
			Absolute benefit: 6.3% hr: 0.64 (*p<*0.0001)		Absolute benefit: 2.7% hr: 0.66 (*p=*0.0115)	
Joint analysis 19 (ncctg N9831, nsabp B-31; 3-Year median follow-up)		3968	85.9	73.1	92.6	89.4
			Absolute benefit: 13% hr: 0.48 (*p<*0.00001)		Absolute benefit: 3.2% hr: 0.65 (*p=*0.0007)	
bcirg 006^20^ (3-Year median follow-up)	ac→th vs. ac→t	3222	83	77	92	86
			Absolute benefit: 6%>br>hr: 0.61 (*p<*0.0001)		— a hr: 0.59 (*p=*0.004)	
	tcbh vs. ac→t		82	77	91	86
			Absolute benefit: 5% hr: 0.67 (*p=*0.0003)		— a hr: 0.66 (*p=*0.017)	

a Absolute benefit value not available.

hera = Herceptin Adjuvant Trial; hr = hazard ratio; ncctg = North Central Cancer Treatment Group; nsabp = National Surgical Adjuvant Breast and Bowel Project; bcirg = Breast Cancer International Research Group; ac = doxorubicin–cyclophosphamide; t = docetaxel.

**TABLE III:** Trastuzumab (h) adjuvant trials: cardiac safety

Trial	Arm	Baseline lvef (%)	Severe heart failure a (%)	Asymptomatic lvef declines b (%)	h discontinued c (%)	h never started c (%)	Cardiac death (n)
hera^18^,^24^	Chemo	≥55	0	0.5	4.3	na	1
	Chemo + h		0.60	3			0
nsabp-31^25^,^26^	ac→p	≥50	0.9^d^				1
	ac→ph	(lln)	3.8^d^				0
ncctg N9831^25^	ac→p	≥50	0.3 d	14.2	18.9 e	6.7	1
	ac→p→h	(lln)	2.5 d				1
	ac→ph		3.5 d				0
bcirg 006 20,27	ac→t	≥50	0.4	10			0
	ac→th	(lln)	1.9	18	na	2.2	0
	tcbh		0.4	8.6		0	0

a New York Heart Association grade iii or iv and decrease in left ventricular ejection fraction of 10 percentage points or more from baseline and to under 50%; does not include death 24.

b New York Heart Association grade i or ii and decrease in left ventricular ejection fraction of 10 percentage points or more below baseline and to under 50% 24.

c Because of cardiac problems.

d Cumulative incidence.

e Asymptomatic decline in left ventricular ejection fraction (14.2) plus heart failure or other adverse cardiac effect (4.7).

lvef = left ventricular ejection fraction; hera = Herceptin Adjuvant Trial; Chemo = chemotherapy; na = not available; nsabp = National Surgical Adjuvant Breast and Bowel Project; ac = doxorubicin–cyclophosphamide; p = paclitaxel; lln = lower limit of normal; ncctg = North Central Cancer Treatment Group; bcirg = Breast Cancer International Research Group; t = docetaxel; cb = carboplatin.

**TABLE IV:** Trastuzumab (h) adjuvant trials: relative risk (rr) of serious, life-threatening, or fatal cardiac events 28

Trial	Treatment		Control		rr
	(n)	(%)	(n)	(%)	
hera^18^	10	0.6	1	0.1	9.97
Joint analysis 19 (ncctg N9831, nsabp B-31)	51	3.1	5	0.3	10.38
bcirg 006 20					
ac→th	20	1.9	4	0.4	5.0
tcbh	4	0.4	4	0.4	1.0

hera = Herceptin Adjuvant Trial; ncctg = North Central Cancer Treatment Group; nsabp = National Surgical Adjuvant Breast and Bowel Project; bcirg = Breast Cancer International Research Group; ac = doxorubicin–cyclophosphamide; t = docetaxel; cb = carboplatin.

**TABLE V:** Five-year cumulative incidence of cardiac events in the National Surgical Adjuvant Breast and Bowel Project B-31 trial 26

Years post day 1, cycle 5	Cumulative incidence of cardiac events (%)		At risk (n)
	Arm 1 a (ac→t)	Arm 2 (ac→th)	
1.0	0.5	3.3	1678
2.0	0.6	3.6	1271
3.0	0.9	3.8	907
4.0	0.9	3.8	569
5.0	0.9	3.8	186

a Arm 1 events among crossover patients censored.

ac = doxorubicin–cyclophosphamide; t = docetaxel; h = trastuzumab.

**TABLE VI:** Recommendations for continuation or withdrawal of trastuzumab therapy in asymptomatic patients based on serial measurements of left ventricular ejection fraction (lvef) a

Relationship of lvef to lln	Asymptomatic decrease in lvef from baseline		
	≤10 Percentage points	10–15 Percentage points	≥15 Percentage points
Within radiology facility’s normal limits	Continue trastuzumab	Continue trastuzumab	Hold trastuzumab and repeat muga or echo after 4 weeks
1–5 percentage points below lln	Continue trastuzumab b	Hold trastuzumab and repeat muga or echo after 4 weeks b,c	Hold trastuzumab and repeatmuga or echo after 4 weeks c,d
≥6 percentage points below lln	Continue trastuzumab and repeat muga or echo after 4 weeks d	Hold trastuzumab and repeat muga or echo after 4 weeks c,d	Hold trastuzumab and repeatmuga or echo after 4 weeks c,c

a Based on National Surgical Adjuvant Breast and Bowel Project B-31 trial protocol 33. Modified to include recommendations for cardiology consultation or treatment of cardiac dysfunction (or both) when appropriate, as indicated in the subsequent footnotes.

b Consider cardiac assessment and initiation of angiotensin converting-enzyme inhibitor therapy.

c After two holds, consider permanent discontinuation of trastuzumab.

d Initiate angiotensin converting-enzyme inhibitor therapy and refer to cardiologist. lln = lower limit of normal; muga = multiple-gated acquisition scan; echo = echocardiography.

**TABLE VII:** Initiating heart failure medication

Medication	Starting dose 21 (mg)	Target dose 21,a(mg)	Suggested titration plan b
ace inhibitors			Increase the dose at 1- to 2-week intervals Monitor renal function and electrolytes weekly or biweekly Maintain blood pressure in normal range Try to reach target dosage in 4 weeks
Captopril	6.25–12.5 ×3 daily	25–50 ×3 daily	
Enalapril	1.25–2.5 ×2 daily	10 ×2 daily	
Ramipril	1.25–2.5 ×2 daily	5 ×2 daily	
Lisinopril	2.5–5 ×1 daily	20–35 ×1 daily	
Beta-blockers c			
Carvedilol	3.125 ×2 daily	25×2 daily	
Bisoprolol	1.25 ×1 daily	10 ×1 daily	

a The target dose should be either the dose used in large-scale clinical trials (listed here), or a lesser but maximum dose tolerated by the patient.

b Initiation of pharmacotherapy for trastuzumab-related cardiotoxicity must be carried out on an accelerated schedule, because the normal titration schedules can take several months to reach optimal therapeutic dosage. The titration plan given here was suggested by the panel, in the absence of clinical data on the use of these medications in trastuzumab-related cardiotoxicity.

c Patients in New York Heart Association (nyha) class i or ii can safely be initiated and titrated with a beta-blocker by non-specialist physicians. Beta-blocker therapy for patients in nyha class iii or iv should be initiated by a specialist experienced in heart failure management and titrated in the setting of close follow-up (such as can be provided in a specialized clinic, if available) 21.

## Appendix A HEART FAILURE AND THE NEW YORK HEART ASSOCIATION FUNCTIONAL CLASSIFICATION

Heart failure 21–23 is a complex clinical syndrome in which abnormal heart function results in, or increases the subsequent risk of, clinical symptoms and signs of low cardiac output or pulmonary or systemic congestion (or both) 22. Heart failure may be the result of any number of cardiac disorders, but most patients with heart failure experience symptoms because of an impairment of left ventricular (lv) myocardial function. Left ventricular dysfunction begins with some injury to, or stress on, the myocardium; it is generally progressive, even in the absence of additional insults to the heart.

The progressive nature of lv dysfunction may be described in terms of cardiac remodelling: over time, the chamber becomes less ovoid and more spherical; it dilates and hypertrophies. Initially compensatory, these changes eventually increase diastolic stiffness and wall tension. Hemodynamic stresses on the walls of the heart increase and mechanical performance decreases. Because not all patients have volume overload at the time of initial or subsequent evaluation, the term “heart failure” is now preferred over the older term “congestive heart failure.”

Two classes of agents have become the recommended cornerstone of therapy to delay or halt progression of cardiac dysfunction and to improve mortality: angiotensin converting-enzyme (ace) inhibitors and beta-blockers. There is compelling evidence that ace inhibitors should be used to inhibit the rennin–angiotensin system in all heart failure patients with lv systolic dysfunction, whether they are symptomatic or not. The Canadian Cardiovascular Society recommends that ace inhibitors be used in all asymptomatic patients with a lvef below 35% and in all patients with symptoms of heart failure and a lvef below 40%.

Beta-blockers are a major advance in the treatment of heart failure. Together with ace inhibitors, beta-blockers are now established as routine therapy in patients with lv systolic dysfunction. The Canadian Cardiovascular Society recommends that all heart failure patients with a lvef below 40% receive beta-blocker therapy.

The Canadian Cardiovascular Society recommends the New York Heart Association (nyha) functional classification as a simple, validated measure of the clinical severity of heart failure 21. The nyha classification describes four grades of heart failure:

- No symptoms
- Symptoms with ordinary activity
- Symptoms with less than ordinary activity
- Symptoms at rest or with any minimal activity

Common clinical presentations of heart failure include dyspnea, orthopnea, paroxysmal nocturnal dyspnea, fatigue, weakness, exercise intolerance, dependent edema, cough, weight gain, abdominal distension, nocturia, and cool extremities.

## Appendix B CHEMOTHERAPY-RELATED CARDIOTOXICITY

Chemotherapy-related cardiotoxicity has been a concern since the early 1970s, when anthracyclines were first shown to be associated with cumulative, dose-related cardiotoxicity 29. Anthracycline-related cardiotoxicity has, for many years, been the “model” for all forms of cardiotoxicity that reduce left ventricular ejection fraction (lvef). However, it has become clear that trastuzumab-related cardiotoxicity does not fit that model: the mechanisms and clinical effects are distinctly different 15,30.

Anthracycline-related cardiotoxicity is largely caused by free radical–induced oxidative stress to cardiac muscle cells. Anthracycline-related cardiotoxicity is cumulative and dose-related, and it results in structural damage to myocytes. The clinical features of heart failure may take months or years to become evident, and although the condition is usually responsive to medical therapy, the underlying damage is largely irreversible. The myocardium pre-exposed to an anthracycline remains more susceptible to subsequent cardiovascular stressors, including hypertension and the effects of trastuzumab- related cardiotoxicity 15,31.

Trastuzumab-related cardiotoxicity is mediated by interruption of the normal her2 signalling pathway in the heart, which maintains normal growth, repair, and survival of cardiomyocytes. Trastuzumab- related cardiotoxicity is not dose-related and appears to be largely reversible 15,30. Evidence from the Breast Cancer International Research Group 006 study showed that the global changes in lvef that occurred with adjuvant trastuzumab therapy recovered to baseline in women receiving the non-anthracycline docetaxel–carboplatin–trastuzumab regimen, but the National Surgical Adjuvant Breast and Bowel Project B-31 and Breast Cancer International Research Group 006 studies demonstrated incomplete lvef recovery in the cohorts that received sequential anthracycline–trastuzumab therapy 20,25–27.
